# Personal Goals, Barriers to Self-Management and Desired mHealth Application Features to Improve Self-Care in Multi-Ethnic Asian Patients with Type 2 Diabetes: A Qualitative Study

**DOI:** 10.3390/ijerph192215415

**Published:** 2022-11-21

**Authors:** Sungwon Yoon, Yu Heng Kwan, Jie Kie Phang, Wee Boon Tan, Lian Leng Low

**Affiliations:** 1 Health Services and Systems Research, Duke-NUS Medical School, Singapore 169857, Singapore; 2 Centre for Population Health Research and Implementation, SingHealth Regional Health System, Singapore 828815, Singapore; 3 Department of Pharmacy, National University of Singapore, Singapore 119077, Singapore; 4 Department of Internal Medicine, Singapore Health Services, Singapore 168753, Singapore; 5 Population Health & Integrated Care Office (PHICO), Singapore General Hospital, Singapore 168753, Singapore; 6 Department of Family Medicine and Continuing Care, Singapore General Hospital, Singapore 169856, Singapore; 7 Post-Acute and Continuing Care, Outram Community Hospital, Singapore 168582, Singapore; 8 Family Medicine Academic Clinical Program, SingHealth Duke-NUS, Singapore 168753, Singapore

**Keywords:** diabetes mellitus, self-management, mobile health application, personal goal, barriers

## Abstract

(1) Introduction: The ubiquity of mobile phones suggests the potential of mobile health applications to reach patients with type 2 diabetes and engage them to improve self-care. This study aimed to explore personal goals, barriers to self-management and desired mobile health application features to improve self-care among multi-ethnic Asian patients with type 2 diabetes. (2) Methods: We conducted semi-structured interviews with patients with type 2 diabetes (*n* = 29). Patients were recruited from a multi-disciplinary center for diabetes and metabolism in Singapore, using a purposive sampling strategy. Various visual materials, collated from existing mobile health application features, were used to facilitate the discussion. Interviews were transcribed verbatim and thematically analyzed. (3) Results: A total of 29 patients participated in 11 focus group discussions or one-on-one interviews. Personal goals for self-management were centered around short-term outcome expectancy, such as better glucose control and a reduced number of medications. Self-management was hampered by competing priorities and limited healthy food options when at work, while a lack of tailored advice from healthcare providers further diminished competence. The desired mobile health app features to improve self-care behaviors included quantifiable goal-setting, personalized nudges based on tracked data, built-in resources from credible sources, in-app social support through virtual interaction with peers and healthcare providers, technology-driven novel data logging and user-defined nudges. (4) Conclusions: We identified a set of app features that may foster motivation to engage in lifestyle modification for patients with T2DM. The findings serve to inform the design of artificial intelligence-enabled mobile health application intervention aimed at improving diabetes self-care.

## 1. Introduction

Type 2 diabetes mellitus (T2DM) is a debilitating chronic disease with lifelong progression. Globally, there were 437.9 million prevalent cases of T2DM in 2019 [1]. In Singapore, where this study was conducted, the prevalence of T2DM among adults aged 18–69 was 14.2%, surpassing the global average (9.3%), and it is estimated to reach 25% in 2050 [2]. Over time, poorly controlled diabetes may lead to life-threatening health problems and complications such as visual loss, amputation, neuropathy, end-stage renal disease and cardiovascular disease [3,4]. Indeed, Singapore has one of the highest incidence rates of T2DM-related end-stage renal failure [5] and amputation [6] in the world, with 49% of patients with T2DM developing microvascular complications [7]. Inadequate diabetic control also places enormous strain on healthcare systems, and brings about lost productivity [8]. The growing social and economic burden associated with poorly controlled T2DM underlines the significance of early intervention.

Self-management through lifestyle modification has been found to be as effective as pharmacological treatment in improving health outcomes of patients with T2DM [9,10]. However, traditional care models may have limited time and resources to monitor and intervene in patients’ lifestyle factors [11]. Mobile health applications (mHealth apps) are increasingly used to foster patients’ motivation for lifestyle modification, and thus improve the self-management of T2DM [12,13]. Systematic reviews and meta-analyses showed that the management of type 2 diabetes through mHealth interventions has been successful in achieving improvements in clinical outcomes [14,15]. Despite this, engagement with mHealth apps among patients with T2DM is crucial for the success of mHealth. The literature suggests that multiple factors influence engagement with mHealth, including patients’ motivation [16] or knowledge [17] to change health behavior, attitude to self-management [18] and the sub-optimal usability of apps [19]. These factors underscore the importance of understanding end users’ perspectives, such as their goals [20,21] and barriers to self-management [22], when developing mHealth apps and features.

To date, several studies have described the needs and preferences of patients with T2DM and factors that could encourage engagement with mHealth apps to support self-management [17,18,23]. For example, people with T2DM would prefer an app to address the practical aspects of diabetes self-management and to reduce the cognitive burden of self-management [24]. However, very few studies have been conducted among patients with T2DM in the context of Asian healthcare and cultural settings. It has been recommended that app features be tailored to the cultural norms and values of users to enhance the acceptability, adherence, and effectiveness of interventions [25,26,27]. Perspectives of patients are crucial to the co-development of mHealth features aimed at enhancing self-management skills and fostering patients’ motivations for continued use [28]. Therefore, building on the existing literature, this study aimed to explore personal goals, barriers to self-management, and views of a mHealth app and its features among multi-ethnic Asian patients with T2DM.

## 2. Materials and Methods

### 2.1. Study Design and Participant Recruitment

This study used a qualitative research method involving focus group discussions (FGD) and in-depth interviews. We have adopted the phenomenological approach to illuminate the challenges of self-management within the healthcare system and social context from the patient’s own perspectives. The phenomenological approach was useful for bringing to the fore the experiences of patients and shared perceptions of mHealth features [29]. Participants were recruited from an integrated, multi-disciplinary center for diabetes and metabolism in Singapore. Singapore is a multi-ethnic city-state located in Southeast Asia. The diabetes center coordinates the delivery of diabetes care within regional hospitals and national specialty centers by combining clinical care and serves as a platform for comprehensive patient care, education and prevention, as well as the management of complications. Eligible participants included those who were 40 years old and above and had the diagnosis of T2DM for more than one year. Patients who presented diabetes-related complications or gestational diabetes were excluded. The study team purposively recruited patients in terms of gender, age and ethnicity from the waiting area of the center to ensure maximum variability of experience and opinions. Written consent was obtained for participation in the study and audio-recording of the interviews. The audio recordings were anonymized prior to transcription to protect the participants’ identities. This study was approved by the SingHealth Centralized Institutional Review Board (2019/2468).

### 2.2. Data Collection

We conducted FGDs with consenting participants from November 2019 to January 2021. The number of participants per FGD ranged from 2 to 6. Owing to scheduling difficulty, two in-depth interviews were conducted separately. A semi-structured topic guide was developed and piloted before the interviews. The main topics included personal goals for diabetes management, factors influencing good diabetes self-management, perceived usefulness of mHealth apps and features that might be useful for improving self-management (Supplementary Text S1). During the interview, visual materials of app features, gathered from existing mHealth apps for T2DM self-management, were presented to facilitate the discussion. The FGDs and in-depth interviews were carried out by an interviewer (WT) who was trained in qualitative research and did not have personal relationships with the participants. We originally planned to conduct physical face-to-face interviews. However, due to the safe distancing measures enforced during the COVID-19 pandemic, we conducted interviews via online video conferencing after the 4th in-person FGDs. After obtaining consent in-person at the outpatient clinic, the study team contacted the participant through text or phone call to arrange the interviews. Similar to in-person sessions, participants were reminded a week and a day before the interview sessions to reduce non-attendance rates. Participants were informed during consent-taking that they would be required to leave their video on during the interview to allow the interviewer to observe any non-verbal cues and to increase sense of participation. Field notes were taken to supplement the interviews. The FGDs and in-depth interviews lasted an average of 120 min and 45 min, respectively. Repeat interviews were not carried out. Transcripts were not returned to participants for comment and/or correction.

### 2.3. Data Analysis

All interviews were audio-recorded and transcribed verbatim. To fully immerse ourselves in the data, we listened to the audio recordings and read through the transcripts to correct any errors in the initial transcribing. Transcripts were then thematically coded using an inductive approach focused on the participants’ interpretations, perceptions, and the ways in which they make sense of the topic. Two coders (SY, JK) read each transcript line by line and coded independently. The coders engaged in an iterative process of reviewing the codes and categories. Discrepancies were resolved through consensus meetings involving all study team members. Emerging themes were continually refined and classified until no new themes emerged from the data. Thematic saturation was reached at the 8th FGDs (*n* = 24), and we conducted 1 additional FGD and two additional interviews to ensure that the point of information redundancy was achieved. No participant checking was carried out for this study. NVIVO 12 was used to store and manage the data. Reporting in this study was based on the consolidated criteria for reporting qualitative research (Table S1) [30].

## 3. Results

### 3.1. Participant Characteristics

A total of 55 eligible patients were invited and 29 patients consented to take part in the study. Reasons for refusal included being busy, no interest and unwillingness to use online conferencing tools for FGDs. Among the participants, the mean age was 57.8 years old (age range: 40–79 years old). About half (55.2%) were males, 62.1% (*n* = 18) were Chinese, and 58.6% (*n* = 17) were employed at the point of recruitment (Table 1 and Table S2).

We conducted nine focus groups and two one-one-one interviews, which yielded important themes related to personal goals, barriers to self-management, and desired mHealth application features in order to improve self-care (Table 2).

### 3.2. Personal Goals for Diabetes Management

#### 3.2.1. Desire to Achieve Good Glycemic Control and Lower the Number of Medications

While personal goals for diabetes management did vary across patients, variations appeared to reflect the individual patients’ preferences for short-term outcomes versus long-term gains. The personal goals were not necessarily mutually exclusive, yet they indicated patients’ personal values. More than half of the participants expressed their desire to achieve good glycemic control and lower the number of medications, based on the presence and absence of symptoms, the need to use multiple medications for chronic co-morbidities and knowledge of the HbA1C reading.

*“I’d like to keep the reading as low as possible. I mean ideally anything below 7 is good for me.”*

(Patient #18, F, 54, Indian)

*“My goal is to reduce the number of the medications, because I’ve got a long list of medications. The other day I went to ask for prescription refill. The nurse looked at me and said I got 3 pages of medications. Hopefully all these can be reduced.”*

(Patient #2, M, 69, Chinese)

#### 3.2.2. Wishing to Avoid Negative Events

Some participants expressed their goal for diabetes management to be avoiding any adverse outcomes arising from the condition. “Foot ulcer” and “diabetic retinopathy” were frequently cited by participants to reflect their concerns about diabetic complications.

*“I’m trying to manage it [diabetes], trying to accept it, but my goal is that I do not want to succumb to complication of diabetes. Definitely not my foot, definitely not my eyes.”*

(Patient #8, F, 55, Malay)

Participants saw their goal for diabetes management to be long-term, and wished to stay healthy in later years of life. This goal was strongly underpinned by their desire to be independent. A particular emphasis was placed on the avoidance of dependency on family for caregiving.

*“Medical cost here is not low, so you do not want to end up being a burden onto your family. Also, caregiving is not that easy. For me, that is important. So, my goal is you don’t want to end up being a burden [to family]. You want to be able to move and do things on your own.”*

(Patient #23, M, 46, Chinese)

#### 3.2.3. Long-Term Gains through Improved Quality of Life

When asked how personal goals were communicated with healthcare professionals, most did not have much experience of setting a specific goal and reflecting on progress together with their providers. For the minority of participants, they felt that they should continue behaving as they did (i.e., adhere to medication regimen) to attain the goal. A handful of participants mentioned that clinical goals should be determined by healthcare providers.

*“I have not really discussed with my doctor on my personal goal. The consultation is short so, not much time for discussion. It [consultation] is about confirming my [sugar] level and telling me the treatment plan. It is more of a tick box type of thing.”*

(Patient #011, M, 62, Malay)

### 3.3. Barriers to Diabetes Self-Management

Barriers surrounding diabetes self-management were largely categorized into three main areas—diet, physical activity and medication.

#### 3.3.1. Diet

Across the three areas, diet was the most salient issue raised by the participants. A majority of participants reported difficulty in complying with regular meal timing due to the nature of their jobs, which involved shift work or irregular work hours. Limited healthy food options also emerged as a barrier to self-management for those who were in the workforce. Some participants described their attempt to adjust portions of unhealthy foods in order to prevent the overconsumption of nutrient-poor food choices.

*“It’s a bit difficult with your diet because when you work, you tend to go eat at a hawker center, which does not have many healthy options. I tell my endocrinologist that sometimes it’s very difficult as a diabetic patient because in hawker center, you have nothing that is low in carbs, low GI. So, you need to eat something anyway with portion control, like half of the carbs of whatever you eat.”*

(Patient #9, M, 50, Chinese)

Another important theme was related to the generic advice given by the healthcare professionals during patient encounters in a healthcare setting. Approximately half of the participants pointed out that standard clinical recommendations were considered blind to the realities of patients’ lives. Negative outcomes or no improvement tended to engender frustration and diminish patients’ trust in healthcare advice and competence.

*“I consulted a dietician and she said, ‘you have to take only this portion, nothing more.’ When I had my meals, I strictly followed the portion recommended by the dietician. What happened was I fainted you know? I really fainted. I work in the supermarket, and I was unable to stay all day with that portion.”*

(Patient #5, F, 56, Chinese)

#### 3.3.2. Physical Activity

For physical activity, despite the general understanding of the health benefits of exercise, participants recounted difficulties in engaging in a sustained physical activity due to physical limitations, such as existing illness or injuries. Work responsibilities or family commitments were mentioned by the majority, prioritizing them over physical activity.

*“So, there’s this 1 h workout which is very good, so every week I always go there for exercise. But during the exercise, I fell down and my bone was broken, after that I stopped the exercise. Now I’m too apprehensive to exercise.”*

(Patient #4, F, 65, Chinese)

*“…there was a period when I could find time to exercise, but after being more involved in work, of course the frequency to exercise has been reduced, and that has caused a spike again in the [glycemic] reading.”*

(Patient #25, F, 47, Indian)

#### 3.3.3. Medication

For medication, participants stated that their work schedule, especially for those engaged in shift work, impeded medication adherence. Forgetfulness was predominantly cited by most participants, resulting in suboptimal medication adherence.

*“Especially when I go out, sometimes I forget to take my medicine. If I go out to eat, then I feel oh did I skip my medicines?”*

(Patient #26, F, 54, Indian)

### 3.4. Desired App Features Useful for Diabetes Self-Management

Participants were asked about the value and usefulness of various mHealth app features that may enable them to improve their self-management of T2DM.

#### 3.4.1. Timely Nudges

By and large, most participants liked the *nudge* function. It was commonly viewed that nudges would be useful for individuals who had the need to remember multiple medications or who had a tendency to forget daily tasks, such as going out for exercise. Some participants mentioned that although similar tools are already available (e.g., mobile phone reminders, which can perform a similar function as the app), nudges personalized to individual patients could improve compliance and hence establish healthy habits.

*“I guess we, as diabetic patients, have an issue of compliance with medication and all that kind of thing, so I guess timely cues would actually enable compliance.”*

(Patient #9, M, 50, Chinese)

*“If you have an app that tells you ‘at lunchtime you should eat this’, then you know what to choose for a meal. The app can also prompt you at 7 pm, ‘you should now walk 1000 steps’. Then you go and walk 1000 steps. This is good because you have a personal coach supporting you daily.”*

(Patient #28, M, 60, Indian)

However, too many nudges were deemed annoying. More than half noted that nudges for food intake, medication and exercise should be personalized and customizable, because otherwise they would be a mere annoyance.

*“I have no problem sleeping, although I do not sleep early, I normally sleep at 11.30 pm, so it’s already like a lifestyle habit, and I do not want to have too many cues. Beep, you exceeded your food intake. Beep, go to sleep now. Beep, take your medicine. I think it’s becoming a nuisance to me.”*

(Patient #14, F, 54, Malay)

#### 3.4.2. Tracking and Personalized Guidance Based on Logs

Participants highly valued a *tracking* feature to track their activity, including sugar levels, medication adherence and exercise, based on logging. It was commonly viewed that tracked data would enable them to learn about progress and increase self-awareness. They stated that self-awareness would allow for better motivation to change lifestyle behaviors.

*“The most important thing is monitoring. You monitor your own behavior and that’s how you can improve. Because you will see there’s a red dot [in HbA1C level] to show that you have not improved, you’ll understand and try to bring it down to green you see.”*

(Patient #29, M, 60, Indian)

In addition to the tracking feature, personalized *guidance* based on one’s inputs was a very positively favored feature. Personalized guidance was particularly valued by one-third of the participants, who felt little or no improvement in their T2DM condition despite adherence to standard recommendations from healthcare professionals. Participants highlighted that they were motivated to follow healthy activities, but did not feel successful in reaching the goal. They liked to receive personalized coaching and real-time feedback tailored to individual inputs with specific personal plans.

*“There are certain features that I find useful. One is the personalized advice. It will be good if the app tells me whether I am doing right or I need to do certain excise to lose fat, or it may be the particular food that increases my sugar level…I feel I do everything I’m supposed to do, but still my sugar level doesn’t change. So, it is important to know how to achieve my goal.”*

(Patient #21, F, 40, Chinese)

Despite the value of tracking and personalized guidance, several participants found that consistent logging could be burdensome, as it may take time and effort to record lifestyle habits.

*“It [logging] could be a bit unfair. I don’t think people have time to sit and log all that information daily. I don’t think I will log every time, like I had a cup of coffee at 12 pm and I had another cup at 2 pm and log…I know it is for your own health, but it just won’t work because I have other important things to do.”*

(Patient #25, F, 47, Indian)

#### 3.4.3. In-App Resources

Around two-thirds of the participants indicated that *in-app resources* would be useful for improving knowledge of healthy lifestyles. They wanted to receive customized suggestions for alternative diet and exercise based on their personal profile (e.g., age, logging) and preferences.

*“It would be good if the app can provide advice on appropriate type of exercise or diet in view of the [health] problem that I have. I’m doing it [exercise] in my own way, but there might be something that the app could help me how to go about doing your daily routine.”*

(Patient #1, F, 79, Chinese)

The resources should be credible and medically accurate; participants noted that while there was an abundance of information online, they were unsure about the reliability of the information.

*“I can easily go to the internet to check exactly how to manage my condition because there’s so much information on the internet. But you don’t know whether the information is reliable. If there’s this app that guides you with steps verified by professionals or doctors, that will be great. Then, we can safely follow the advice.”*

(Patient #12, F, 60, Malay)

#### 3.4.4. App-Based Peer Support and Built-in Chat with Health Coaches

Lastly, a handful of participants desired an *app-based peer support group* or a *built-in chat with health professionals*. It was viewed that these features could serve as a source of motivation and a platform to share experiences among patients with similar challenges.

*“Peer support is a source of motivation. You can use the app to chat ‘so how do you maintain your sugar level? How do you go about doing it?’ So, it’s not just one-way system. I mean if the app is just technical, it’s dead and the information you get from it is very fixed. But when you chat with a fellow diabetic patient, you can learn and support each other.”*

(Patient #17, F, 47, Chinese)

## 4. Discussion

This study sought to explore personal goals, factors hampering self-management and useful app features that can empower patients with T2DM to improve self-care among multi-ethnic Asian patients.

Our findings show that personal goals for T2DM self-management were primarily centered around short-term outcome expectancy [31], such as better glucose control and reduced medication intake, while a minority described long-term gains such as better quality of life and prevention of future complications. Despite various goals, participants’ accounts suggest that personalized goal-setting and the monitoring of progress on goal attainment were rare in routine care. This is in line with prior studies that show patients’ lack of experience with goal-setting [32]. The literature suggests that setting personal goals and negotiating behavior changes are critical to enhancing the patient’s commitment to diabetes self-care [11,33]. However, time pressures in consultations and the lack of readiness of patients were reported as major challenges to adopting such process [32,34,35,36]. Patients need ongoing support and mHealth has the potential for facilitating patient engagement in this respect. A systematic review demonstrates that defining a goal into quantifiable measurements through mHealth can have a substantial impact on health outcomes for patients managing T2DM [37]. Likewise, a meta-analysis highlights that mHealth interventions using “action planning” and “self-monitoring of outcome(s)” were particularly effective in reducing HbA1c [38]. Therefore, goal-setting via mHealth can be usefully incorporated into routine care to supplement existing health service delivery and improve the engagement of patients in diabetes self-management.

We found that across three main areas of T2DM self-management (i.e., diet, physical activity and medication), healthy diet is the most difficult challenge for patients with T2DM. Participants commonly attributed this to the failure to follow regular mealtimes and limited healthy food options when eating out during working hours. This finding resonates with studies that found that having meals out of the home was one of the most frequently cited problems that prevented patients with T2DM from controlling their condition [39,40]. This finding underlines the importance of prevention strategies at the policy and community level, such as increase in healthier food options and improved food labeling. Singapore has a long tradition of street vendors and open-air complexes selling a variety of food [41], and local eating venues such as hawker centers play a more dominant role than Western fast food chain [42]. At the individual level, recommendations for diet should be tailored to local food consumption patterns to advise healthier food choices that are commonly available near workplaces. Related to this, our participants felt the health advice they received from healthcare providers was too generic and not as useful as it could be. Patients expected more customized recommendations based on personal circumstance and needs. In light of the time pressures during consultation, as discussed earlier, personalized nudges via mHealth apps in response to patient-reported data might have the potential to improve patients’ capability to self-manage their condition while mitigating the workload of healthcare providers. Evidence attests to the effectiveness of personalized advice on health outcomes [37], and hence more research is needed on how best to frame targeted messages and deliver them at the right time.

Participants in our study were generally positive about the use of the mHealth app for diabetes self-management. They saw the mHealth app as a platform to improve lifestyle habits and learn how to manage their conditions. In particular, participants desired a *nudge* function that prompts them to take medication at the specific times as prescribed, since forgetfulness was the primary barrier to optimal medication adherence. There was also consensus that knowing one’s history of physical activity and other health-related data through a *tracking* function was seen as beneficial and motivating. Another feature that was highly valued by participants was *in-app resources*. This finding is in line with prior research that certain mHealth app features play a vital role in improving behavior modifications and health outcomes in T2DM patients. For example, mobile app-assisted interventions that use tracking showed higher adherence to self-management behaviors [43]. Similarly, educational content through the in-app resource function was found to have a positive effect on improving awareness, although a reduction in HbA1c was not consistently observed [44]. Despite the utility of these mHealth features, participants’ accounts indicated that frequent nudges and extensive routines of data entry for various activities were seen as ineffective, presenting an impediment to the use of mHealth apps. It is therefore essential to address these barriers to improve uptake. For instance, having user-defined nudges based on individual needs as opposed to notifications grounded in algorithms alone might lead to better patient experiences and engagement. Since wearable options are conducive to tracking physical activity data, photo-enabled food diaries [45] or speech-based logging for food consumption and medication intake [46] could provide an additional novel opportunity to reduce user burdens.

The literature demonstrates that social support presents an important psychosocial element for adherence to self-care in T2DM patients [47,48]. Social support allows for a sense of belonging, emotional exchanges and acceptance that increases patients’ ability to cope with stress associated with self-management. Indeed, our findings show that in-app peer support and health coaching by professionals would be an appealing feature for patients. The literature indicates that two-way communication via mHealth between a healthcare provider and patient may be useful for glucose monitoring and patient adherence [37,49]. Additionally, emotional, motivational and practical assistance offered by peer supporters (e.g., peer educators, patient navigators) has been shown to improve patient outcomes, such as reductions in HbA1c levels [50]. However, interventions leveraging virtual peer interactions to foster healthy behavior using a mHealth app are largely absent [51]. Therefore, more research is warranted to evaluate the potential of app-based communication features that facilitate the sharing of self-care experiences, concerns and challenges.

### Strengths and Limitations

This study adds important evidence to the potential for mHealth-based self-management and the utility of app features from the perspectives of multi-ethnic Asian patients with T2DM. However, the findings of this study should be considered in light of a few limitations. Owing to the qualitative nature of the study involving a small sample, the findings may not represent the views of all patients with T2DM. However, we strived to purposively recruit a diverse range of patients to maximize perspectives. We recruited patients from a public healthcare institution, and therefore we may have missed the views of patients from private healthcare institutions. We used visual materials to facilitate the discussion of an mHealth app and its features. Therefore, feedback from participants might have been confined to the features presented during the interview. In addition, remote interviewing via online conferencing following the COVID-19 pandemic might have excluded some participants with low digital literacy. It is possible that patients who agreed to participate may have higher levels of digital literacy, which may have influenced the findings on mHealth. Moreover, we did not focus on exploring patients’ views of minimally invasive glucose measuring tools and other glycemic control technologies in this study, which can be explored in a future study. We were not able to capture the data in terms of how experiences of self-management and perceptions of mHealth might have differed between identities (gender, age and ethnicity profiles), which could have afforded richer insights into the perceptions of these sub-groups. Lastly, around half of the patients refused participation in this study, and this might have introduced a selection bias. It is possible that those who participated had fewer barriers to self-care and more positive attitudes toward mHealth than those who did not.

## 5. Conclusions

Emerging mHealth technologies alter the way patients with T2DM track their data and improve self-management. This study provides important insights into the design of an mHealth app through a better understanding of barriers to self-management and the perceived values of specific app features aimed at improving diabetes self-care. Our study highlights the importance of tailoring mHealth apps to local cultures, such as incorporating local food options and recommendations. The desired app features to improve self-care behaviors include quantifiable goal-setting, personalized nudges, resources from credible sources, in-app support through virtual interaction with peers and healthcare providers, technology-driven novel data logging and user-defined reminders. Our findings serve to inform the development of artificial intelligence-enabled mHealth intervention for patients with T2DM.

## Figures and Tables

**Table 1:** Participant characteristics (*n* = 29).

	*n*	%
Number of focus groups	9	
Number of one-one-one interviews	2	
Age (years)		
mean (SD)	57.8 (9.38)	
Range	40–79	
Gender		
Male	16	(55.2)
Female	13	(44.8)
Ethnicity		
Chinese	18	(62.1)
Malay	6	(20.7)
Indian	5	(17.2)
Education		
Primary or Lower	1	(3.4)
Secondary	15	(51.7)
Tertiary or above	13	(44.8)
Marital Status		
Single/Never married	3	(10.3)
Married	23	(79.3)
Divorced/Widowed	3	(10.3)
Employment Status		
Employed	17	(58.6)
Unemployed	3	(10.3)
Retired	9	(31.0)

**Table 2:** Summary of themes and sub-themes.

	Theme/Domain	Subtheme
Personal goals	Desire to achieve good glycemic control and lower the number of medications	Having good glycemic control
		Reducing the number of medications
	Wishing to avoid negative events	Circumventing death, prompted by witnessing the suffering of family and friends
		Keeping away from T2DM complications
	Long-term gains through improved quality of life	Staying well in later years of life
		Maintaining independence so as not to be a burden to family
Barriers to self-management	Diet	Unable to comply with regular meal timing
		Limited healthy food options at work
		One-size-fits-all advice felt to be inadequate engendering loss of motivation
	Physical activity	Existing ailments hinder exercise
		Insufficient time to exercise due to competing priorities
	Medication	Work schedules hampering adherence
		Tendency to forget regular regimen
App features	Timely nudges (cues)	Nudges felt to be helpful for enhancing compliance
		Utilizing nudges as a form of personal coaching
		Too many nudges perceived as a nuisance and desire for customization
	Tracking and personalized guidance based on logs	Tracking allows one to learn progress and enhance self-awareness
		Personalized feedback viewed helpful particularly for those who followed generic recommendations but did not work
		Daily logging can be a burden
	In-app resources	Desire to receive customized suggestions for alternative exercise and diet
		Credible and clinically accurate information seen as crucial
	App-based peer support and built-in chat with health coaches	App-based activity as a source of motivation and information-sharing

## Data Availability

The data presented in this study are available on request from the corresponding author. The data are not publicly available due to ethical concerns.

## Supplementary Materials

The following supporting information can be downloaded at: https://www.mdpi.com/article/10.3390/ijerph192215415/s1, Supplementary Text S1: Interview guide, Table S1: COREQ, Table S2: Demographic details of participants.
