# Supplementary material for: Personal Goals, Barriers to Self-Management and Desired mHealth Application Features to Improve Self-Care in Multi-Ethnic Asian Patients with Type 2 Diabetes: A Qualitative Study
Source: Int J Environ Res Public Health. 2022 Nov 21;19(22):15415. doi: 10.3390/ijerph192215415 (PMC9692780; doi:10.3390/ijerph192215415)
Supplement: Supplementary file 1 [file ijerph-19-15415-s001.zip › ijerph-1977952-supplementary.pdf]

International Journal of
*Environmental Research and Public Health*

MDPI

**Supplementary Materials**

**Supplementary Text S1.** Interview guide.

Interview Guide

1. Can you share some information about diabetes you live with and problems you have in managing your condition(s)?

[Probe]

a. DM specific medical management (symptom control, medication, treatment follow-up)
b. Role management (work, family, function, daily life issues)
c. Emotional management (stress, frustration, fear of starting on insulin)
d. Other issues (financial, cultural)

2. What is your overall goal with diabetes management? What do you wish to achieve?

2-1. What is your idea of ideal diabetes management?

[Probe]

a. Symptom (e.g., relieving certain symptoms)
b. Function (e.g., improving daily function, quality of life)
c. Physical consequences (e.g., avoid amputation, glaucoma…explore misconception)
d. Others (e.g., not to spend money on medication or dialysis)

2.2. How confident are you in your ability to achieve your goals with diabetes management? What made you think so?

[Probe]

a. Knowledge and Skills
b. Perseverance (goal-specific)

4. What are some ways you have tried to take good control of your conditions to meet your goal(s) mentioned just now?

**Discuss the following components separately*

- *Physical activity*
- *Diet*
- *Medication*
- *Sleep*
- *Others*

[Probes]

a. What prompted those attempts?
b. What have been helpful?

c. What influences how to take care of your condition each day?
d. What are some things that could help you to self-manage your condition? (e.g. family support, use of technological device, support from healthcare providers)

4. Tell me about things you tried but were unsuccessful.

[Probes]
a. What problems did you have?
b. What types of barriers have you experienced?
c. For how long have you been aware of these barriers?
d. Have you tried addressing these barriers before? If yes, how? If not, why?

5. How important is it for you to take good control of your conditions? And why?

** take the level of knowledge of participants into consideration.*

[Probes]
a. Competing priorities: how important is it to take good control of your conditions compared to other areas in your life (e.g., taking care of children, job requirements)?
b. What would help you to stay motivated to follow a lifestyle that can manage your conditions?
c. Are you aware of the recommended standards to manage your conditions (e.g., minimum of 150 minutes of moderate-vigorous physical activity per week)?
d. To what extent are you motivated to meet these standards?

**6.** Let's talk about your experience with the health care system and health care providers related to your conditions.

[Probe]
a. Overall, how do you feel about health care systems and providers when it comes to managing your chronic conditions?
b. What would you say about your healthcare providers in terms of motivating you to manage and take good control of your conditions? To what extent they have influenced life-style change and effective diabetes self-management and care?
c. What are your current healthcare needs and issues?
d. Which one(s) are more important than others and why?
e. How do you think such needs could be met?

7. What is your experience of using mobile applications and fitness trackers to manage your conditions?

**Selected visual materials are used to facilitate the discussion*

[Probe]
*(For those who have used before/continue to use or stopped using it)*

a. Which app or trackers have you used?
b. How did you learn about the app/trackers? Where did you learn it from? (e.g. family, peers, provider, online articles, online reviews, etc.)
c. For how long and how often have you been using the app/tracker?
d. To what extent are you dependent on the app/trackers?
e. What aspects of app/trackers worked well (or helped better manage your conditions) or did not work well? (e.g., self-monitoring, tailoring goals, dealing with complications, reminders, incentives, default options, etc)
f. Why did you stop using an app? How long have you used? When did you last use the app/tracker?
g. What can help you continue to use the app?

*(For those who have not used)*
What were the reasons for your decision not to use such apps or trackers?

8. We are aiming to develop a mobile app that can provide you with automated personalised coaching and reminders in a timely manner to help manage your conditions. If such app is available, how likely you will use it? If yes, why? If not, what are some of the things that keep you from using it?

**Discuss specifically under different dimensions*
*Operational*
*Psychological/emotional*
*Others*

[Probe]
a. What sort of features would be useful and relevant to you?
   [Internal, for facilitator]
- Linked to electronic medical records
- Ability to review blood test results
- Clinic appointment schedule and reminder
- Online support groups
- Virtual Chat
- Video Conferencing
- Medical report on your health status/progress
- Blood glucose/blood pressure readings
- Others

b. Given your schedule, what is the kind of reminders and timing of the reminders that will work best for you?
c. How can the app 'motivate' one to improve his/her diabetic conditions? Which aspect is more important to you and why?
   [Internal, for facilitator]
- Gamifications
- Rewards / Incentives
- Framing
- Feedback

- Prompting / Reminders
- Self-education/information videos & readings
- Problem solving
- Setting Defaults
- Walkthroughs
- Chatbot

8.1. Revisiting to your overall goals for the diabetes management earlier, in what ways the new app (e.g., features) can help you to overcome potential short-term obstacles (e.g. making an effort to find time to exercise, temptation to eat unhealthy foods) to achieve your long-term goals mentioned above?

9. Is there anything else you would like to add on the topics we have discussed today?

Thanks for your participation. Your comments will be very helpful to us and the mobile app we are planning to develop for people with diabetes.

**Table S1.** Consolidated criteria for reporting qualitative research (COREQ): 32-item checklist.

| Item Number | Guide Questions | Reported on |
|---|---|---|
| **Domain 1: Research team and reflexivity** | | |
| *Personal Characteristics* | | |
| 1. Interviewer/facilitator | Which author/s conducted the interview or focus group? | In Methods |
| 2. Credentials | What were the researcher's credentials? E.g. PhD, MD | PhD MPH |
| 3. Occupation | What was their occupation at the time of the study? | Academic faculty in the University and staff at a medical research center |
| 4. Gender | Was the researcher male or female? | Females |
| 5. Experience and training | What experience or training did the researcher have? | Training in health services and public health |
| *Relationship with participants* | | |
| 6. Relationship established | Was a relationship established prior to study commencement? | No relationship was established prior to study commencement. |
| 7. Participant knowledge of the interviewer | What did the participants know about the researcher? e.g. personal goals, reasons for doing the research | Participants were aware of the primary purpose of the study (Methods) |
| 8. Interviewer characteristics | What characteristics were reported about the inter viewer/facilitator? e.g. Bias, assumptions, reasons and interests in the research topic | Interviewers have experience in conducting qualitative interviews (in Methods). |
| **Domain 2: study design** | | |
| *Theoretical framework* | | |

| | | |
|---|---|---|
| 9. Methodological orientation and Theory | What methodological orientation was stated to underpin the study? e.g. grounded theory, discourse analysis, ethnography, phenomenology, content analysis | Inductive thematic analysis (in Data analysis) |
| *Participant selection* | | |
| 10. Sampling | How were participants selected? e.g. purposive, convenience, consecutive, snowball | Purposive sampling was employed (in Data collection) |
| 11. Method of approach | How were participants approached? e.g. face-to-face, telephone, mail, email | Face-to-face (in Data collection) |
| 12. Sample size | How many participants were in the study? | 29 (in Results) |
| 13. Non-participation | How many people refused to participate or dropped out? Reasons? | 26 because they were busy or not interested (in Data collection) |
| *Setting* | | |
| 14. Setting of data collection | Where was the data collected? e.g. home, clinic, workplace | In person or via online video conferencing such as Zoom (data collection). |
| 15. Presence of non-participants | Was anyone else present besides the participants and researchers? | No |
| 16. Description of sample | What are the important characteristics of the sample? e.g. demographic data, date | Characteristics of the sample were described in Results and Table 1. |
| *Data collection* | | |
| 17. Interview guide | Were questions, prompts, guides provided by the authors? Was it pilot tested? | In data collection and supplementary material |
| 18. Repeat interviews | Were repeat interviews carried out? If yes, how many? | No |
| 19. Audio/visual recording | Did the research use audio or visual recording to collect the data? | Yes, in data collection |
| 20. Field notes | Were field notes made during and/or after the interview or focus group? | Field notes were made. |
| 21. Duration | What was the duration of the inter views or focus group? | Between 45 – 60 min (in section 2.2). |
| 22. Data saturation | Was data saturation discussed? | Yes (in data analysis) |
| 23. Transcripts returned | Were transcripts returned to participants for comment and/or correction? | Transcripts were not returned to participants. |
| | **Domain 3: analysis and findings** | |
| *Data analysis* | | |

| | | |
|---|---|---|
| 24. Number of data coders | How many data coders coded the data? | 2 (in data analysis). |
| 25. Description of the coding tree | Did authors provide a description of the coding tree? | Yes, but not presented in the manuscript. |
| 26. Derivation of themes | Were themes identified in advance or derived from the data? | Themes were derived from the data (in data analysis). |
| 27. Software | What software, if applicable, was used to manage the data? | NVivo was used (in data analysis). |
| 28. Participant checking | Did participants provide feedback on the findings? | Participants were not provided with the findings. |
| *Reporting* | | |
| 29. Quotations presented | Were participant quotations presented to illustrate the themes/findings? Was each quotation identified? e.g. participant number | Quotations were presented and participant number provided. |
| 30. Data and findings consistent | Was there consistency between the data presented and the findings? | Consistency was checked (data analysis). |
| 31. Clarity of major themes | Were major themes clearly presented in the findings? | Major themes were clearly presented (results). |
| 32. Clarity of minor themes | Is there a description of diverse cases or discussion of minor themes? | Minor themes and diverse cases were described (results). |

**Table S2.** Demographic details of respondents (N = 29).

| Participant No | Gender | Age | Ethnicity | Highest Level of Education | Marital Status | Employment Status | FGD Session |
|---|---|---|---|---|---|---|---|
| 01 | Female | 79 | Chinese | Diploma/Professional Qualifications | Married | Retired | 1 |
| 02 | Male | 69 | Chinese | Secondary | Married | Retired | 1 |
| 03 | Male | 63 | Chinese | Diploma/Professional Qualifications | Single | Retired | 1 |
| 04 | Female | 65 | Chinese | Tertiary or above | Single | Employed | 2 |
| 05 | Female | 56 | Chinese | Primary or lower | Widowed | Employed | 2 |
| 06 | Male | 74 | Chinese | Tertiary or above | Married | Retired | 2 |
| 07 | Female | 61 | Chinese | Tertiary or above | Divorced | Employed | 3 |
| 08 | Female | 55 | Malay | Tertiary or above | Married | Employed | 3 |
| 09 | Male | 50 | Chinese | Tertiary or above | Married | Employed | 3 |
| 10 | Female | 53 | Chinese | Tertiary or above | Married | Unemployed | 3 |
| 11 | Male | 62 | Malay | Secondary | Married | Unemployed | 4 |
| 12 | Female | 60 | Malay | Secondary | Divorced | Employed | 4 |
| 13 | Male | 68 | Malay | Secondary | Married | Retired | 4 |
| 14 | Female | 54 | Malay | Secondary | Married | Unemployed | 4 |
| 15 | Male | 63 | Chinese | Tertiary or above | Married | Retired | 4 |
| 16 | Male | 66 | Chinese | Secondary | Single | Retired | 4 |
| 17 | Female | 47 | Chinese | Secondary | Married | Employed | 5 |
| 18 | Female | 54 | Indian | Tertiary or above | Married | Employed | 5 |
| 19 | Male | 62 | Chinese | Diploma/Professional Qualifications | Married | Retired | 5 |
| 20 | Female | 60 | Indian | Secondary | Married | Employed | 6 |
| 21 | Female | 40 | Chinese | Tertiary or above | Married | Employed | 6 |
| 22 | Female | 41 | Chinese | Tertiary or above | Married | Employed | 7 |
| 23 | Male | 46 | Chinese | Tertiary or above | Married | Employed | 7 |
| 24 | Female | 45 | Chinese | Diploma/Professional Qualifications | Married | Employed | 8 |

| | | | | | | | |
|---|---|---|---|---|---|---|---|
| 25 | Female | 47 | Indian | Tertiary or above | Married | Employed | 8 |
| 26 | Female | 54 | Indian | Diploma/Professional Qualifications | Married | Employed | 9 |
| 27 | Male | 61 | Chinese | Tertiary or above | Married | Retired | 9 |
| 28 | Male | 60 | Indian | Secondary | Married | Employed | IDI 01 |
| 29 | Male | 60 | Indian | Secondary | Married | Employed | IDI 02 |
